# Lines of Zahn in a Patient With Transjugular Intrahepatic Portosystemic Shunt Occlusion From a Tumor Thrombus: A Case Report

**DOI:** 10.7759/cureus.88011

**Published:** 2025-07-15

**Authors:** Sam J King, Clever Nguyen, Mary Beth Brownlee, Richard Kalman

**Affiliations:** 1 Internal Medicine, Jefferson Einstein Philadelphia Hospital, Philadelphia, USA; 2 Physical Medicine and Rehabilitation, Jefferson Einstein Philadelphia Hospital, Philadelphia, USA; 3 Hepatology, Philadelphia College of Osteopathic Medicine, Philadelphia, USA

**Keywords:** alcohol-related cirrhosis, cirrhosis, hcc, hepatocellular carcinoma, portal hypertension, portal vein thrombosis, tips, tips occlusion, transjugular intrahepatic portosystemic shunt, tumor thrombus

## Abstract

Hepatocellular carcinoma (HCC) in patients with alcohol-related cirrhosis is commonly associated with complications such as ascites, variceal bleeding, hepatic encephalopathy, coagulopathy, and portal vein thrombosis (PVT). A significant number of patients with HCC require transjugular intrahepatic portosystemic shunt (TIPS) placement. To date, there have been no reported cases of HCC with tumor thrombus occluding a TIPS. We present the case of a 50-year-old man with alcohol-related cirrhosis, status post-TIPS placement, and unresectable stage IIIA/Barcelona Clinic Liver Cancer (BCLC) C HCC, who had been treated with brachytherapy. He presented with painful abdominal distension. Physical examination revealed large-volume ascites and caput medusae. Diagnostic paracentesis was performed, draining approximately 3 liters of sanguineous ascitic fluid. Laboratory analysis indicated non-neutrocytic, culture-negative transudative hemorrhagic ascites. A computed tomography (CT) scan of the abdomen and pelvis revealed interspersed areas of contrast-enhanced opacification within the TIPS, suggestive of partial TIPS thrombosis. Ultrasound of the liver with Doppler demonstrated slow TIPS flow with reversal of flow throughout the entire portal venous system, overall concerning for shunting of blood away from the TIPS and TIPS malfunction. Interventional radiology was consulted for TIPS revision and thrombectomy. The patient underwent aspiration thrombectomy, balloon venoplasty, and TIPS extension. Post-procedural TIPS venography demonstrated a significantly increased TIPS caliber, improved flow through the TIPS, and reduced portal venous pressures. Microscopic analysis of the evacuated thrombus revealed fragments of HCC with necrosis. Additionally, a rare pathology finding, lines of Zahn, was observed, indicating acute thrombus formation. Given the involvement of the portal vein, the patient was upstaged to American Joint Committee on Cancer (AJCC) stage IIIB/BCLC C. Oncology was consulted, with initial plans for first-line systemic therapy using tremelimumab and durvalumab contingent on clinical improvement and reduction in bilirubin levels below 3 mg/dL. However, given the patient's Child-Pugh Class C (score of 11), the potential benefit from systemic therapy was deemed limited. Outpatient follow-up and treatment reevaluation were planned. On hospital day 9, the patient was discharged in stable condition with oncology follow-up scheduled. This case highlights a rare but serious complication of TIPS placement in patients with advanced HCC, underscoring the importance of close monitoring for TIPS occlusion due to tumor thrombus, which can significantly affect treatment outcomes.

## Introduction

Alcoholic cirrhosis is a significant risk factor in developing hepatocellular carcinoma (HCC) [1]. The presence of HCC with cirrhosis increases the risk of portal vein thrombosis (PVT) compared to cirrhosis alone [2]. PVTs are clinically significant because they can worsen portal hypertension, increasing the frequency and severity of associated sequelae such as ascites, hepatic encephalopathy, and variceal bleeding. Extension of the PVT into the mesenteric venous outflow tract can also result in intestinal ischemia, bowel infarction, and ileus [3]. Treatment of HCC depends on the degree of portal hypertension and functional status according to the Barcelona Clinic Liver Cancer (BCLC) staging system, and modalities include surgical resection, liver transplantation, ablation, transarterial chemo-/radioembolization (TACE/TARE), external beam radiation, and systemic therapy [4]. Transjugular intrahepatic portosystemic shunt (TIPS) placement, in addition to reducing portal hypertension, has also been shown to improve nodule count and overall survival in liver transplant candidates with HCC [5]. Further, cases of HCC structural invasion besides HCC-related PVT have been shown. These include HCC-related bile duct tumor thrombi, hepatic vein tumor thrombi, and regrowth of primary HCC tumor through a trans-tumor TIPS [6,7]. However, to the best of our knowledge, no cases of HCC tumor thrombus burden occluding a TIPS have been reported. We present a unique case of recurrent HCC tumor thrombus causing TIPS occlusion in a patient with aggressive HCC requiring repeat thrombectomy.

## Case presentation

**Figure 1:**
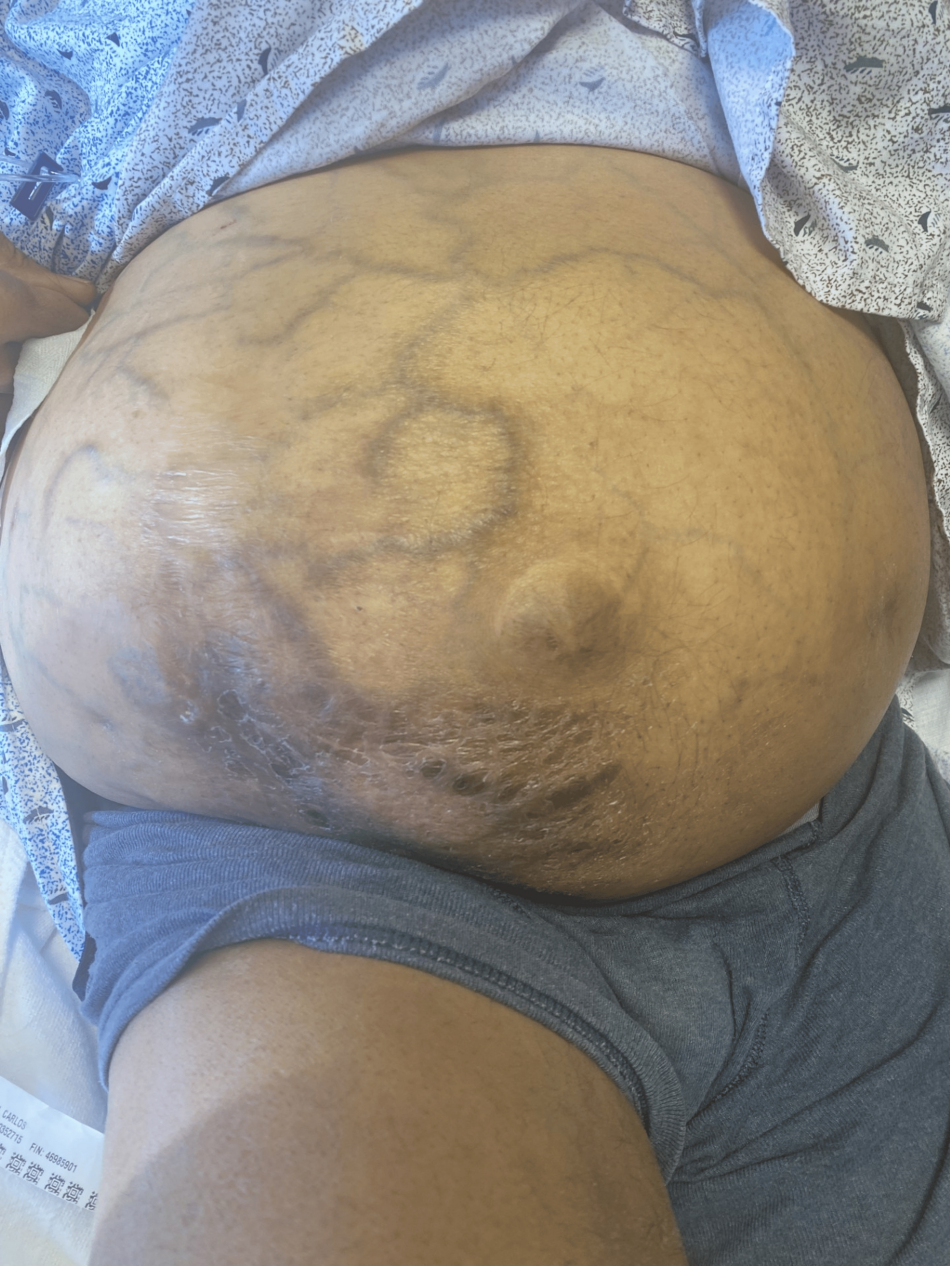
Ectopic varices: caput medusae

A 50-year-old man with a history of alcohol-related cirrhosis, status post-TIPS placement secondary to recurrent variceal bleeding, unresectable stage IIIA/BCLC C HCC treated with locoregional brachytherapy using yttrium-90 (Y90), and atrial fibrillation (not on anticoagulation therapy due to a history of high-risk variceal bleeding) presented with painful abdominal distention. While in the emergency department, an initial examination was performed and revealed significant abdominal distension with positive fluid wave and caput medusae (Figure 1).

**Figure 2:**
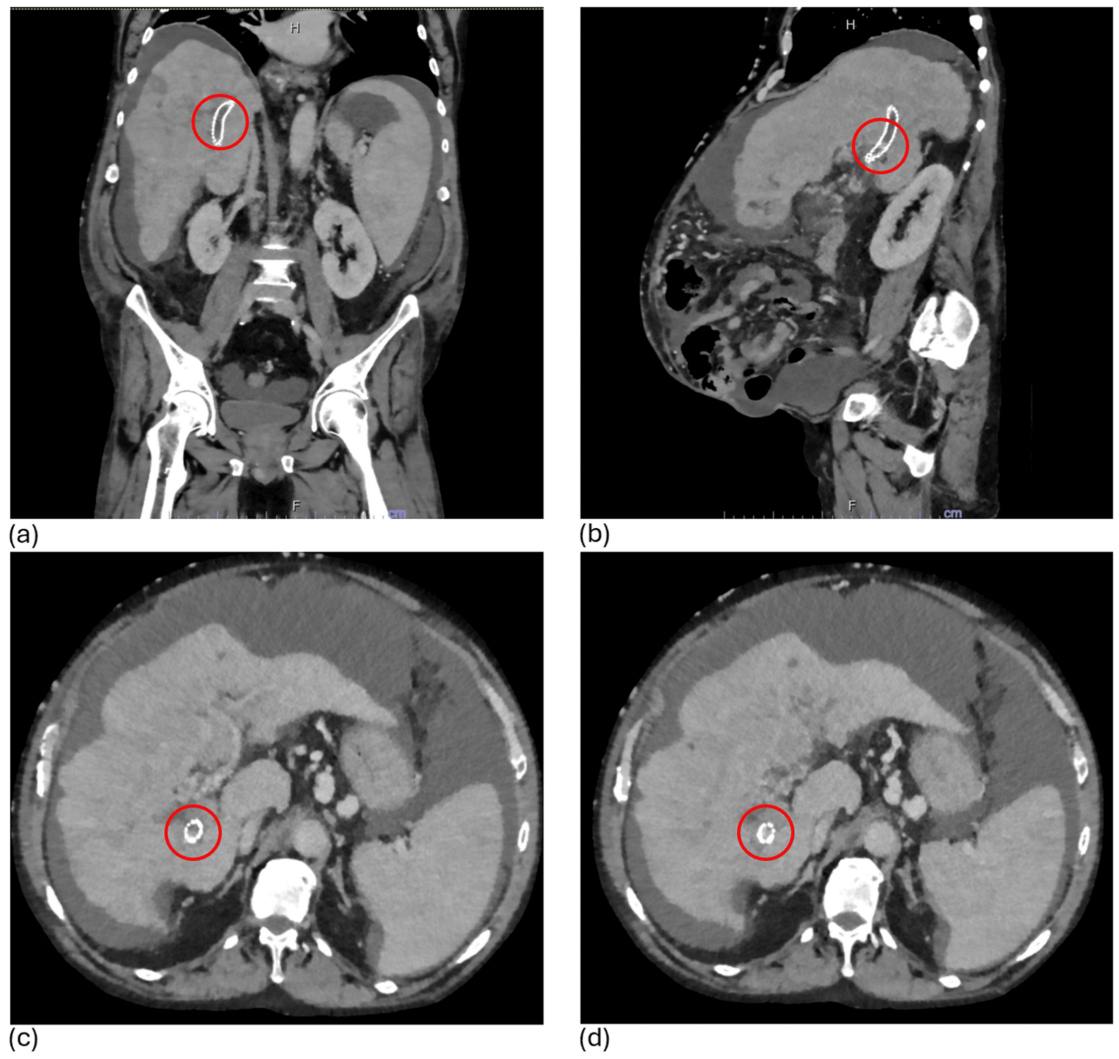
(a) Coronal, (b) sagittal, and (c-d) axial images from CT of the abdomen and pelvis with IV contrast showing intra-TIPS thrombus, stigmata of cirrhosis, and multifocal stage AJCC IIIB/BCLC C HCC Technique: Multidetector helical CT of the abdomen and pelvis was performed. IV contrast: 100 cc Isovue-370 CT: computed tomography; IV: intravenous; TIPS: transjugular intrahepatic portosystemic shunt; AJCC: American Joint Committee on Cancer; BCLC: Barcelona Clinic Liver Cancer; HCC: hepatocellular carcinoma

Laboratory evaluation was significant for anemia with a hemoglobin level of 7.6 g/dL, a normal serum white blood cell count, and an elevated international normalized ratio (INR) of 1.5. A diagnostic paracentesis was also performed, draining approximately 3 liters of bloody ascitic fluid. Laboratory analysis of the peritoneal fluid indicated non-infectious, transudative hemorrhagic ascites. A computed tomography (CT) scan of the abdomen and pelvis was also obtained, demonstrating small areas of intermittent contrast opacification within the TIPS, suggestive of partial TIPS thrombosis, with thrombus burden likely extending from the portal venous end to the hepatic end (Figure 2).

The patient was admitted to the Hepatology/Step-Down Unit for the monitoring of his hemoglobin and further evaluation of his worsening hemorrhagic ascites. 

**Figure 3:**
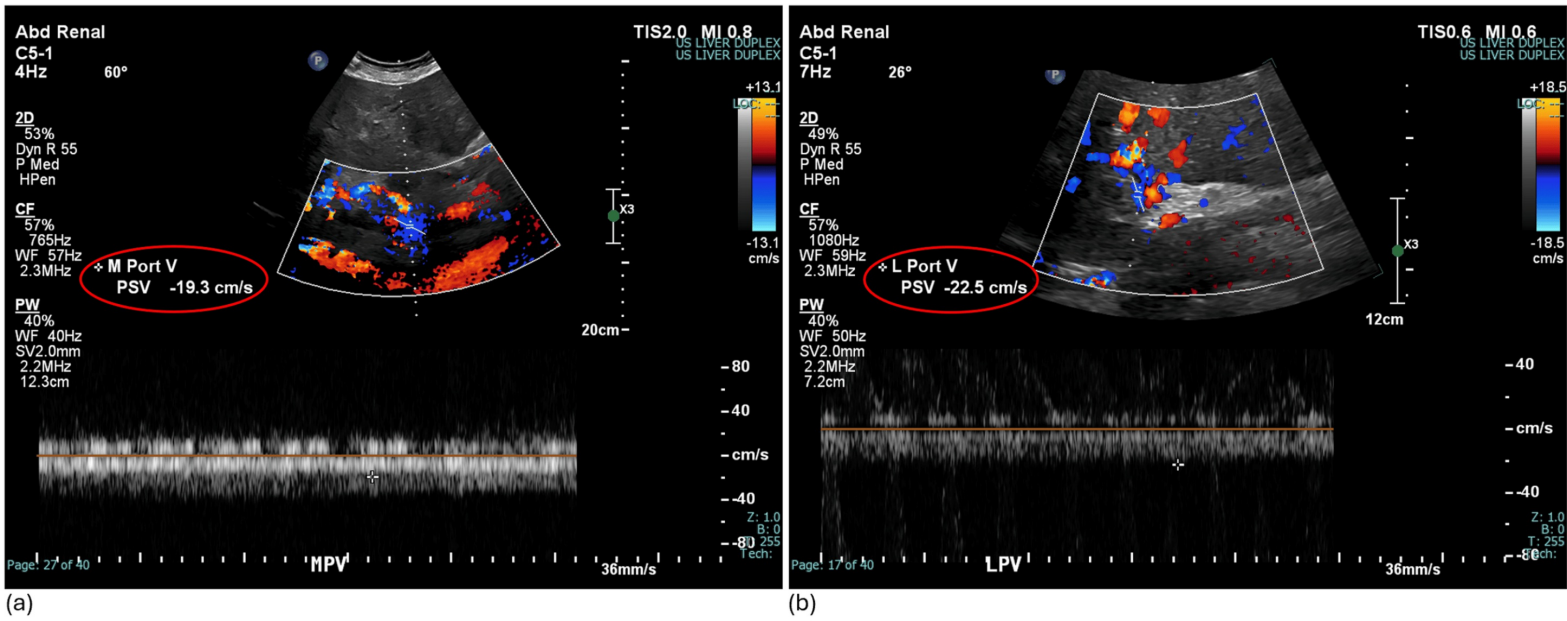
Liver Doppler US showing slow flow throughout the portal venous system including the (a) main portal vein and (b) left portal vein US: ultrasound; PSV: peak systolic velocity; M Port V: main portal vein; L Port V: left portal vein

**Figure 4:**
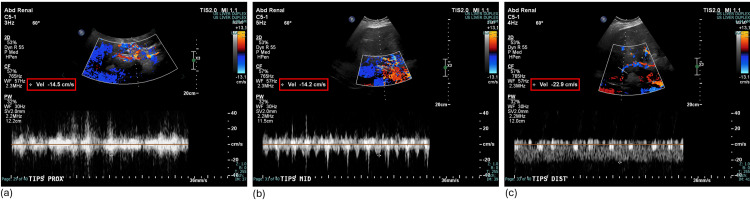
Doppler US showing slow flow through TIPS including (a) proximal TIPS, (b) mid-TIPS, and (c) distal TIPS US: ultrasound; TIPS: transjugular intrahepatic portosystemic shunt

**Figure 5:**
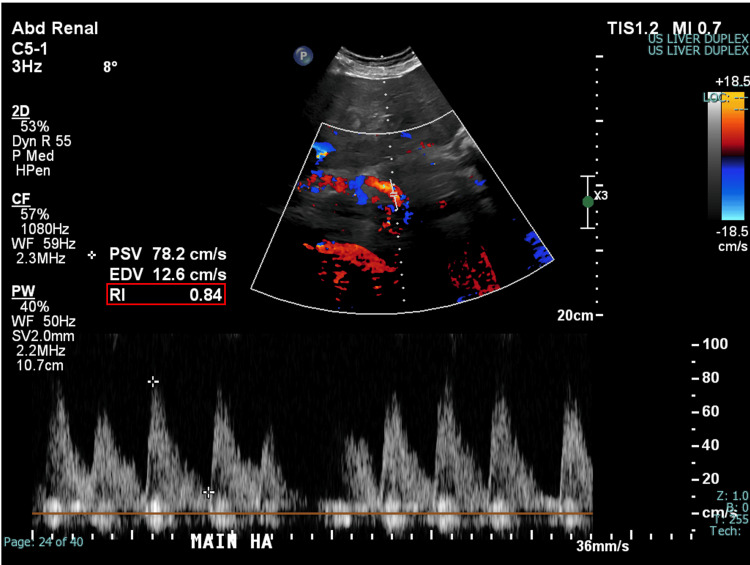
Doppler US of the proper hepatic artery showing elevated resistive index US: ultrasound; Main HA: main hepatic artery (proper hepatic artery); PSV: peak systolic velocity; EDV: end diastolic velocity; RI: resistive index

While on the Step-Down Unit, his hemoglobin was monitored serially and remained stable. A liver ultrasound was obtained showing reversal of flow throughout the entire portal venous system (Figure 3), slow velocities within the TIPS (Figure 4), and an elevated resistive index in the proper hepatic artery (Figure 5). The velocities of each measurement are listed in Table 1 along with the normal values for comparison. Together, these findings were concerning for shunting of blood away from the TIPS and overall TIPS malfunction.

**Table 1:** Key ultrasound findings prior to TIPS revision US: ultrasound; PSV: peak systolic velocity; MPV: main portal vein; LPV: left portal vein; TIPS: transjugular intrahepatic portosystemic shunt; HA: hepatic artery; RI: resistive index

Key ultrasound findings		
Portal venous system Doppler US		
Site	Measured PSV	Normal reference range
MPV	-19 cm/s	20-40 cm/s
LPV	-22.5 cm/s	20-40 cm/s
TIPS Doppler US		
Site	Measured PSV	Normal reference range
Proximal TIPS	14.5 cm/s	90-190 cm/s
Mid-TIPS	14.2 cm/s	90-190 cm/s
Distal TIPS	22.9 cm/s	90-190 cm/s
Main HA Doppler US		
Site	Measured RI	Normal reference range
Main HA	0.84	0.55-0.80

**Figure 6:**
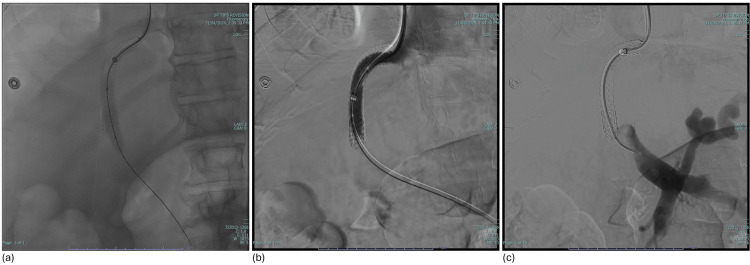
Multi-panel angiography showing (a) balloon venoplasty, (b) improved TIPS flow, and (c) improved portal venous system flow TIPS: transjugular intrahepatic portosystemic shunt

Interventional radiology was consulted for TIPS revision and thrombectomy. The patient subsequently underwent aspiration thrombectomy, balloon venoplasty, TIPS extension, coil embolization of two gastric varices, and an additional paracentesis. Post-procedural TIPS venography demonstrated a significantly increased TIPS caliber, improved flow through the TIPS, and reduced portal venous pressures (from 31 mmHg to 24 mmHg) (Figure 6).

**Figure 7:**
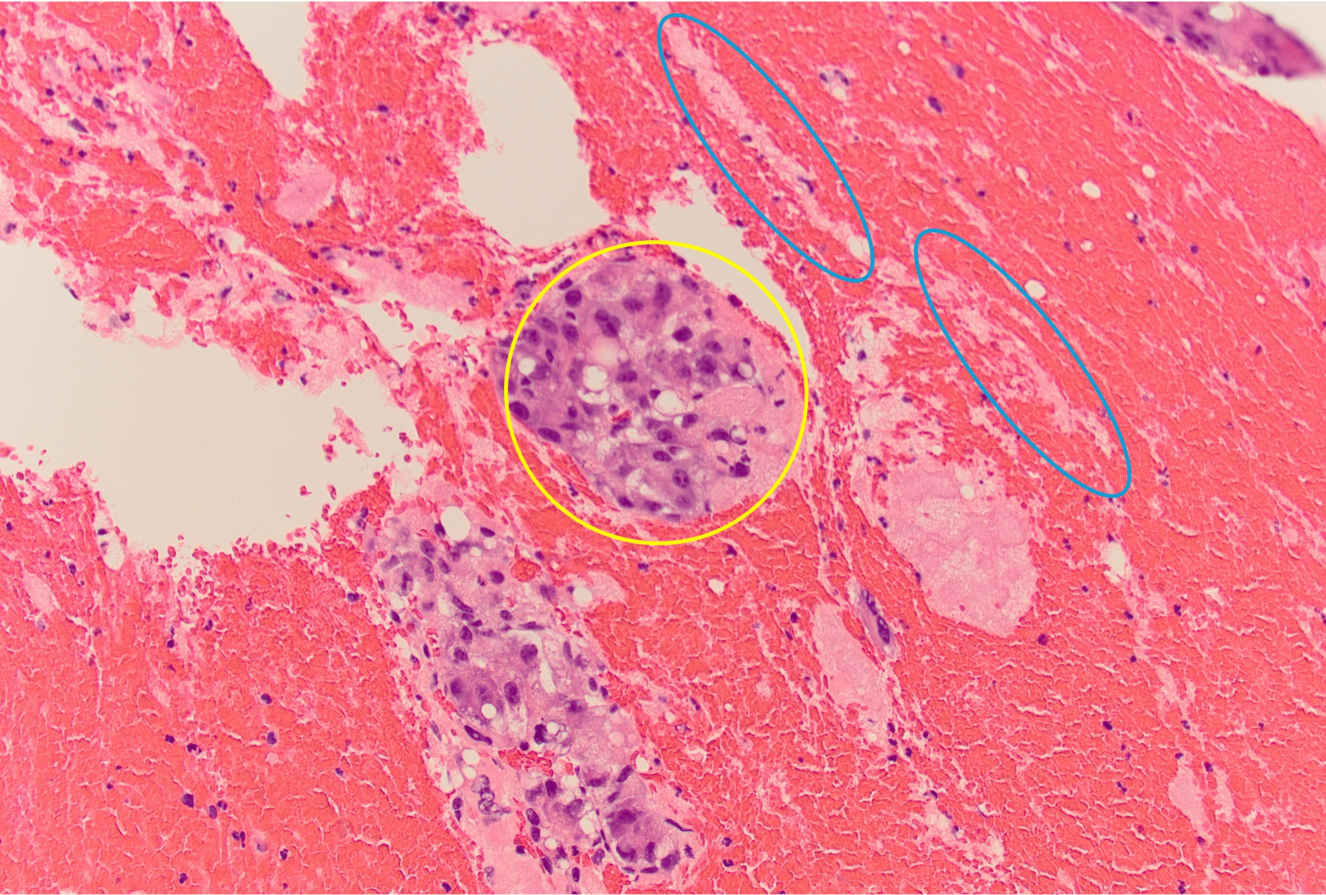
Cross-section of IHC-stained intra-TIPS thrombus showing fragments of necrotic HCC (yellow circle) and lines of Zahn (blue ovals) (darker layers represent erythrocytes, while lighter layers represent fibrin and platelets) IHC: immunohistochemistry; TIPS: transjugular intrahepatic portosystemic shunt; HCC: hepatocellular carcinoma

Pathological analysis of the evacuated thrombus revealed fragments of HCC with necrosis. Additionally, the presence of lines of Zahn was observed (Figure 7), indicative of acute thrombus formation.

Given the involvement of the portal vein, the patient was upstaged to American Joint Committee on Cancer (AJCC) stage IIIB/BCLC C. Oncology was consulted, with initial plans for first-line systemic therapy using tremelimumab and durvalumab contingent on a clinical improvement and reduction in the patient's bilirubin levels to below 3 mg/dL. However, given the patient's Child-Pugh Class C (score of 11), the likelihood of meaningful benefit from systemic therapy was considered low at that time. Outpatient reevaluation of treatment options was planned, and on hospital day 9, the patient was discharged in stable condition with oncology follow-up scheduled for the next day.

## Discussion

Patients with HCC are at a higher risk of developing PVT than patients with cirrhosis. For example, the prevalence of PVT in patients with hepatic cirrhosis has been reported to be between 0.6% and 26%, while that in patients with HCC from 20% to 44% [8,9]. In patients with HCC-associated PVT (also known as portal vein tumor thrombosis or PVTT), the median survival is 2.7 months if untreated [10]. Therefore, patients with PVTT are at a high risk of morbidity and mortality. Further, cases of direct tumor spread occluding TIPS have been described, with reported cases including a handful of patients with TIPS traversing hepatic malignancies [7]. However, to the best of our knowledge, no cases of HCC tumor thrombus occlusion of TIPS have been reported. 

TIPS procedures have been shown to be helpful in patients with HCC. Several retrospective trials have assessed the impact of TIPS on median overall survival in patients with HCC. Five studies comprising a total of 496 patients with HCC who underwent TIPS placement were compared. The median overall survival ranged between 2.5 and 50 months; the magnitude of this range likely represents the high variability between different patient populations. The rate of TIPS malfunction requiring revision ranged between 7.1% and 15.5% [11]. Further, TIPS procedures in patients with HCC can improve access to curative therapies and improve overall survival. In a prospective study of 106 patients with advanced HCC who were receiving tyrosine kinase inhibitor (TKI) therapy and had already received endoscopic intervention plus beta-blockade, TIPS placement was associated with a significant reduction in rebleeding, prolonged survival, and increased adherence to TKI therapy compared to repeat endoscopic intervention plus beta-blockade [12]. However, large-scale prospective studies assessing the role of TIPS in HCC are lacking; therefore, further investigation is required. Such studies could also assess the risk factors for tumor thrombus occlusion of TIPS in patients with HCC and the optimal monitoring strategies for TIPS patency in patients with advanced HCC.

## Conclusions

Our case aims to bring awareness to the rare presentation of invasive HCC causing acute tumor thrombus, subtotal TIPS occlusion, and hemorrhagic ascites in a patient with alcohol-related cirrhosis. TIPS procedures in patients with HCC have been shown to have a high technical success rate and to improve access to curative therapies as well as overall survival. However, as our case illustrates, patients with HCC are at an increased risk of TIPS occlusion due to tumor thrombus formation, and such patients with TIPS occlusion are at an increased risk of morbidity and mortality due to the effects of portal hypertension. Oncologists are responsible for patient follow-up after TIPS placement and must be aware of complications that may occur in the setting of progressive disease.

## References

[1] Huang DQ, Tan DJ, Ng CH (2023). Hepatocellular carcinoma incidence in alcohol-associated cirrhosis: systematic review and meta-analysis. Clin Gastroenterol Hepatol.

[2] Samant H, Asafo-Agyei KO, Kimyaghalam A (2024). Portal vein thrombosis. StatPearls [Internet].

[3] Brown ZJ, Tsilimigras DI, Ruff SM, Mohseni A, Kamel IR, Cloyd JM, Pawlik TM (2023). Management of hepatocellular carcinoma: a review. JAMA Surg.

[4] El Hajji S, Lacotte S, Moeckli B, Cauchy F, Compagnon P, Toso C (2024). Transjugular intrahepatic portosystemic shunt is associated with better waitlist management of liver transplant candidates with hepatocellular carcinoma. Transpl Int.

[5] Tadokoro T, Tani J, Morishita A, Fujita K, Masaki T, Kobara H (2024). The treatment of hepatocellular carcinoma with major vascular invasion. Cancers (Basel).

[6] Wallace M, Swaim M (2003). Transjugular intrahepatic portosystemic shunts through hepatic neoplasms. J Vasc Interv Radiol.

[7] Tripodi A, Anstee QM, Sogaard KK, Primignani M, Valla DC (2011). Hypercoagulability in cirrhosis: causes and consequences. J Thromb Haemost.

[8] Rabe C, Pilz T, Klostermann C, Berna M, Schild HH, Sauerbruch T, Caselmann WH (2001). Clinical characteristics and outcome of a cohort of 101 patients with hepatocellular carcinoma. World J Gastroenterol.

[9] Llovet JM, Bustamante J, Castells A (1999). Natural history of untreated nonsurgical hepatocellular carcinoma: rationale for the design and evaluation of therapeutic trials. Hepatology.

[10] Fichtl A, Seufferlein T, Zizer E (2023). Risks and benefits of TIPS in HCC and other liver malignancies: a literature review. BMC Gastroenterol.

[11] Chen Y, Ma X, Zhang X (2022). Prevention of variceal rebleeding in cirrhotic patients with advanced hepatocellular carcinoma receiving molecularly targeted therapy: a randomized pilot study of transjugular intrahepatic portosystemic shunt versus endoscopic plus β-blocker. Hepatol Int.

[12] Galasso L, Cerrito L, Termite F (2024). The molecular mechanisms of portal vein thrombosis in hepatocellular carcinoma. Cancers (Basel).

